# Mobile genetic elements in shaping *Klebsiella pneumoniae* pathogenicity

**DOI:** 10.3389/fmicb.2025.1730961

**Published:** 2026-01-06

**Authors:** Yanbing Li, Refath Farzana

**Affiliations:** 1 State Key Laboratory of Complex Severe and Rare Diseases, Department of Clinical Laboratory, Peking Union Medical College Hospital, Chinese Academy of Medical Sciences and Peking Union Medical College, Beijing, China; 2 Graduate School, Peking Union Medical College, Chinese Academy of Medical Sciences, Beijing, China; 3 Department of Biology, Ineos Oxford Institute for Antimicrobial Resistance, University of Oxford, Oxford, United Kingdom

**Keywords:** integrative conjugative element, *Klebsiella pneumoniae*, mobile genetic elements, pathogenicity, plasmid, virulence

## Abstract

*Klebsiella pneumoniae* has evolved from an opportunistic pathogen into a formidable global threat, with hypervirulent strains now causing severe infections in healthy individuals and carbapenem-resistant variants achieving mortality rates exceeding 42%. This transformation can be driven by mobile genetic elements including plasmids, integrative conjugative elements (ICE), insertion sequences (ISs), transposons, and integrons. Recent discoveries reveal that these elements employ sophisticated mechanisms: conjugative virulence plasmids dissemination across bacterial populations; ICEs-mediated virulence traits transfer; and hybrid genetic elements simultaneously confer virulence and antimicrobial resistance. Understanding these molecular mechanisms is critical for developing targeted diagnostics and therapeutics that disrupt mobile element mobility, offering promising strategies to combat the convergence of hypervirulence and resistance in this WHO priority pathogen.

## Background: from opportunistic pathogen to superbug, the evolution of *Klebsiella pneumoniae*

*Klebsiella pneumoniae* represents a paradigmatic example of rapid pathogenic evolution in the modern clinical era. Historically recognised as an opportunistic pathogen primarily causing nosocomial infections in immunocompromised hosts, this Gram-negative bacterium has undergone a remarkable transformation that fundamentally challenges traditional concepts of bacterial pathogenicity (Paczosa and Mecsas, 2016). Classical *K. pneumoniae* (cKP) isolates retain this profile, exhibiting high genetic diversity and typically low virulence (Kochan et al., 2023). The pivotal recognition of this evolutionary shift of this organism occurred in 1986 when Liu et al. documented seven cases of invasive *K. pneumoniae* infections manifesting as hepatic abscess and septic endophthalmitis in previously healthy community-dwelling individuals without underlying biliary tract disease. This seminal observation marked the emergence of what would later be characterised as hypervirulent *K. pneumoniae* (hvKp), distinguished by its capacity to cause severe invasive diseases in immunocompetent hosts (Liu et al., 1986).

Subsequent decades witnessed parallel *K. pneumoniae* evolution along two increasingly convergent pathways. The first involves hypervirulence development, characterised by enhanced capsular polysaccharide (CPS) production (mucoid phenotype), iron acquisition systems (aerobactin, yersiniabactin, salmochelin), and systemic infection establishment from initial colonisation sites (Shon and Russo, 2012; Gu et al., 2018). The second pathway encompasses extensive antimicrobial resistance (AMR) acquisition, particularly emergence of carbapenem-resistant *K. pneumoniae* (CRKP) which has been linked to mortality rates as high as 42.86% (Chen et al., 2022). Trait convergence has culminated in carbapenem-resistant hypervirulent *K. pneumoniae* (CR-hvKp), a true “superbug” combining exceptional pathogenic potential with broad AMR profiles (Gu et al., 2018; Roulston et al., 2018). Complexity intensifies through highly clonal populations and persistence of environmental reservoir (hospital surfaces, water systems, medical equipment), facilitating rapid healthcare dissemination (Paczosa and Mecsas, 2016; Farzana et al., 2023).

Given substantial pathogenic potential, rising AMR challenges, and epidemiological significance as both harmless coloniser and pathogen, *K. pneumoniae* has garnered extensive scientific attention. In recognition of these concerns, the 2024 WHO Bacterial Priority Pathogens List (BPPL) formally designated carbapenem-resistant *K. pneumoniae* as the top “critical priority” pathogen, assigning it the highest possible risk score (84%) amongst 24 evaluated bacteria (Sati et al., 2025). This designation reflects its high burden of disease, increasing resistance trends, treatment difficulty, and significant public-health impact globally. Given its substantial pathogenic potential, evolving resistance profiles, and epidemiological relevance, *K. pneumoniae* remains a major threat and therefore continues to warrant focused research and surveillance.

Central to understanding this rapid pathogenic evolution is recognising that the transformative capacity of *K. pneumoniae* can be driven by mobile genetic elements (MGEs). These genetic structures including plasmids, integrative and conjugative elements (ICEs), insertion sequences (ISs), transposons, and integrons, function as horizontal gene transfer vehicles, enabling rapid virulence and resistance trait dissemination across diverse bacterial populations and accelerating bacterial adaptation to selective pressures (Ernst et al., 2020; Farzana et al., 2023).

Despite significant advances in understanding *K. pneumoniae* pathogenicity, critical knowledge gaps remain regarding precise mechanisms governing MGE-mediated virulence acquisition and dissemination. Regulatory networks controlling virulence gene expression, molecular bases of MGE mobility, and factors determining successful horizontal transfer events remain incompletely characterised (Haudiquet et al., 2021).

This review provides comprehensive overview of MGEs associated with *K. pneumoniae* virulence. We focus on mechanisms underlying pathogenic trait transfer between plasmids and chromosomes, and amongst different bacterial strains. By highlighting established and predicted virulence factors and analysing their genetic contexts, this review elucidates MGE roles in shaping *K. pneumoniae* pathogenic potential.

Data systematically collected from eligible studies included: virulence gene location (chromosome or plasmid); MGEs pertinent to virulence, including size and classification (e.g., group for insertion sequences; replicon type for plasmids); bacterial host harbouring virulence genes; sequence type (ST) and K-antigen (capsular type) of the host bacteria; as well as year, country and source of isolation of bacterial host.

## Understanding hypervirulence: genetic determinants in *Klebsiella pneumoniae*

**Figure 1:**
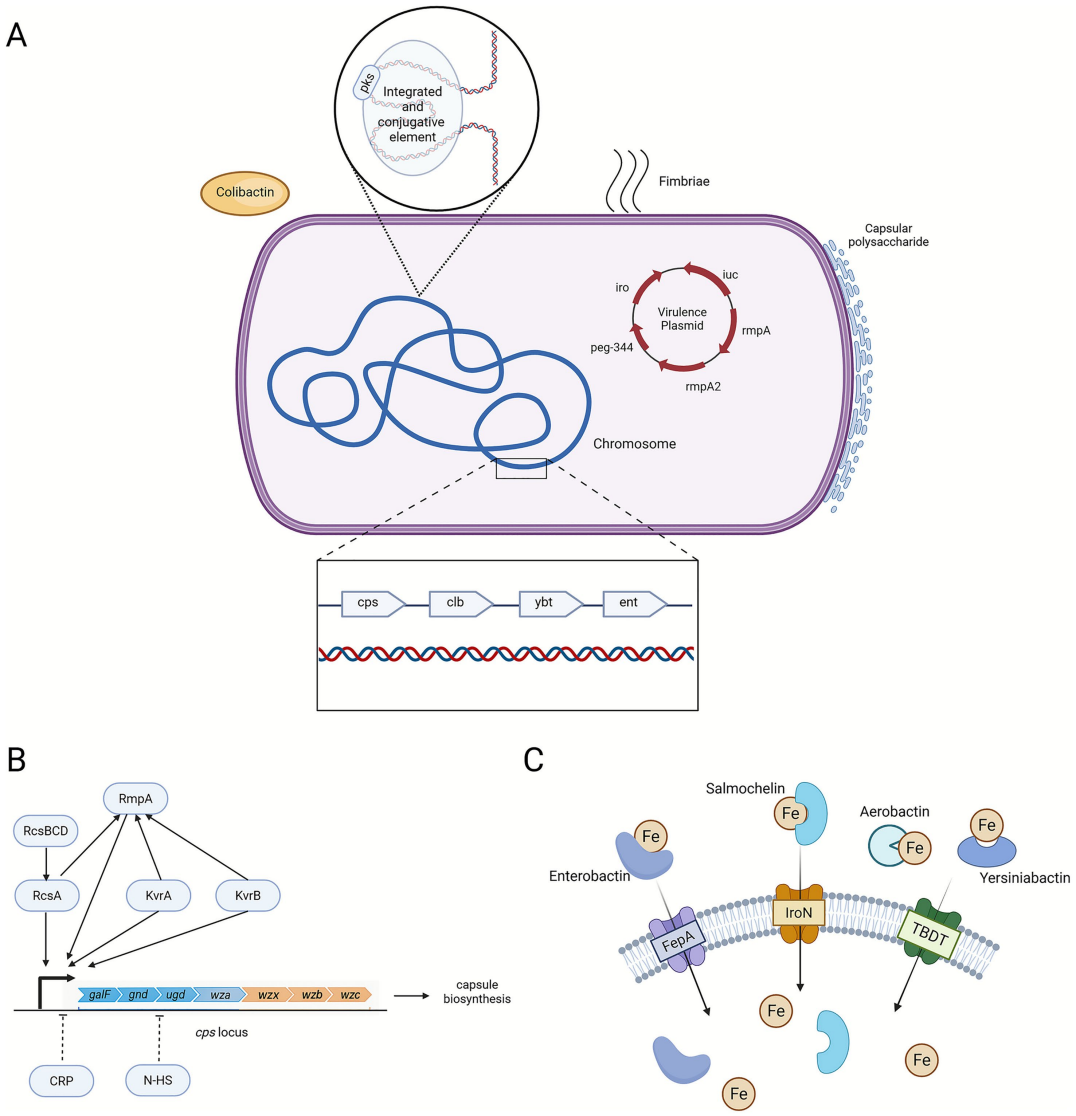
Major virulence factors in *K. pneumoniae*. **(A)** Key virulence genes are distributed across the chromosome and virulence plasmids. Chromosomally encoded siderophores include *ent* and *ybt*, the latter often located on an ICE alongside the *clb* locus responsible for colibactin biosynthesis. Virulence plasmids encode siderophore systems such as *iuc*, *iro,* and regulatory genes like *peg-344*, all contributing to increased pathogenicity. Additional virulence factors, such as fimbriae, also contribute to *K. pneumoniae* virulence. **(B)** Regulatory factors of CPS in *K. pneumoniae*. Solid arrows denote promotion, and dashed arrows indicate repression. Positive regulators, including RmpA, KvrA, KvrB, and the RcsA/RcsBCD phosphorelay system, activate transcription of key capsule biosynthesis genes (*galF*, *gnd*, *ugd*, *wza*, *wzx*, *wzb*, and *wzc*), thereby enhancing CPS production and contributing to the hypermucoviscous phenotype. In contrast, CRP and nutrient- or stress-associated signals (N-HS) act as negative regulators that repress *cps* transcription. **(C)** The siderophore systems enterobactin *(ent)*, yersiniabactin *(ybt)*, salmochelin *(iro),* and aerobactin *(iuc)* are present. Each siderophore binds iron outside the bacterial cell and delivers it to specific outer-membrane receptors. These uptake pathways allow the bacterium to overcome host iron limitation and support its growth and virulence. TBDT: TonB-dependent transporter. The figure is created by BioRender.

Since the discovery of hvKp, how to genetically and phenotypically classify hvKp has become an urgent question. Early investigations identified the mucoid phenotype and siderophore systems as key contributors to hvKp characteristics (Figure 1A) (Nassif and Sansonetti, 1986; Nassif et al., 1989). This review categorise the virulence genes in *K. pneumoniae* according to their mechanisms of action (Table 1).

**Table 1:** Summary of virulence genes in *K. pneumoniae*.

Category	Gene/gene cluster	Function	Phenotypic effect	Genetic location	Representative references
Capsule regulation & CPS biosynthesis	*galF*, *gnd*, *ugd*	Sugar synthesis for CPS precursor formation	Capsule biosynthesis	Chromosomal CPS locus	Xu et al. (2024)
	*wzy*, *wzx*	Capsule polymerisation	CPS chain biosynthesis	Chromosomal CPS locus	Xu et al. (2024)
	*wza*, *wzb*, *wzc*	Capsule transport	Export of CPS to cell surface; *wzc* SNPs increase virulence	Chromosomal CPS locus	Ernst et al. (2020)
	*magA*	K1-specific capsule formation; SNPs increase mortality	Hypervirulence; enhanced serum resistance	Chromosomal (K1)	Fang et al. (2010)
	*rmpA*, *rmpA2*, *rmpB*	Capsule upregulation; hypermucoviscosity	Classic hvKp markers; highly prevalent in K1/K2	Mainly plasmid; occasionally chromosomal	Wacharotayankun et al. (1993) and Hsu et al. (2011)
	*rcsA*, *rcsB*, Rcs phosphorelay	Stress-responsive activation of cps	Increased capsule production	Chromosomal	Meng et al. (2021)
	*kvrA*, *kvrB*, *fur*, *lon*	Capsule regulators	Modulate CPS levels; Fur represses *entC*	Chromosomal	Palacios et al. (2018)
	*CRP*	Global regulator; represses cps	Reduced capsule under CRP activation	Chromosomal	Lin et al. (2013)
	*mlaA*, *PLD*, Mla system	Maintain membrane integrity, indirectly stabilising CPS	Capsule stability	Chromosomal	Dorman et al. (2018)
Iron acquisition systems	Enterobactin (Ent): *entC*, *fepA*	Primary siderophore production & receptor	Iron scavenging; baseline virulence factor	Chromosomal	Baghal et al. (2010)
	Yersiniabactin (*Ybt*)	Siderophore synthesis	Promotes systemic infection	Chromosomal/ICEKp	Schalk (2025)
	Aerobactin (*iucABCD*-*iutA*)	High-affinity siderophore	Strongly associated with hvKp	Plasmid-borne (virulence plasmids)	Holt et al. (2015)
	Salmochelin (*iroBCDN*)	Glycosylated enterobactin	Immune evasion; hvKp marker	Plasmid-borne	Müller et al. (2009)
	*iroN*, *iroB*	Salmochelin uptake and synthesis	Enhanced fitness	Plasmid-borne	Müller et al. (2009)
	*kfu*	Iron uptake system	Fitness and virulence enhancement	Chromosomal	Hsieh et al. (2008)
	*tonB*	Energises siderophore uptake	Iron import	Chromosomal	Hsieh et al. (2008)
	Fur	Ferric uptake regulator	Represses *entC*; modulates siderophore expression	Chromosomal	Yuan et al. (2020)
	RcsAB complex	Regulates *entC* under iron limitation	Activates siderophore genes under stress	Chromosomal	Yuan et al. (2020)
Genotoxin	Colibactin (*pks* island)	Genotoxic compound causing DNA damage	Cell cycle arrest, epithelial disruption; enhances dissemination	Chromosomal (often on ICE)	Choby et al. (2020) and Lu et al. (2017)
Other validated virulence genes	*peg-344*	Biomarker for hvKp; unknown function	Strong hvKp predictor	Plasmid	Bulger et al. (2017)
	*mrk* fimbriae	Type III fimbriae	Adhesion, biofilm formation	Chromosomal	Lai et al. (2003)
	*moaR*, *kva15*	Regulatory proteins	Modulate virulence traits	Chromosomal	Chou et al. (2004) and Tu et al. (2009)
	*kvgAS*	Two-component system	Increases virulence gene expression	Chromosomal	Chou et al. (2004)
	Allantoin metabolism genes	Allantoin utilisation	Contributes to liver infection	Chromosomal	Lai et al. (2003)
	*arcZ* (sRNA)	Represses virulence genes	Post-transcriptional regulation	Chromosomal	Wu et al. (2024)
	*tolB*, *tolR*, *pal*, *lpp*, *ompA*, *waaL*, *nlpI*	Cell envelope integrity	Enhances serum resistance and virulence	Chromosomal	Zhu et al. (2023)

### Essential genes for capsule and hypermucoviscosity production

CPS overproduction represents a critical virulence factor enabling immune evasion and facilitating survival within host environments. Capsule biosynthesis is encoded by the *cps* gene cluster, including genes for sugar synthesis (*galF*, *gnd*, *ugd*), polymerisation (*wzy*, *wzx*), and transport (*wza*, *wzb*, *wzc*) (Xu et al., 2024). Single-nucleotide polymorphisms (SNPs) in *wzc* (Ernst et al., 2020) and K1-specific *wzy* polymerase formerly known as *magA* (Fang et al., 2010) increase mortality in animal models. The *cps* locus is regulated by plasmid-borne and chromosomal factors: the hypermucoviscous phenotype associates with K1 and K2 serotypes through the *rmp* operon (*rmpA*-*rmpC*-*rmpD*) (Walker Kimberly et al., 2019; Walker Kimberly et al., 2020; Wacharotayankun et al., 1993) which are typically plasmid-encoded but also found in chromosome (Hsu et al., 2011). Chromosomal regulators (*rcsA*, *rcsB*, and the Rcs phosphorelay system) respond to stress and upregulate *cps* genes (Meng et al., 2021). Additional chromosomally encoded regulators like *kvrA*, *kvrB*, *fur*, and *lon* modulate capsule expression (Palacios et al., 2018), while cAMP receptor protein (CRP) negatively regulates by repressing *cps* transcription (Lin et al., 2013). Phospholipid transport systems such as Mla system, MlaA, Phospholipase D (PLD) maintain membrane integrity and capsule stability (Lin et al., 2013; Dorman et al., 2018) (Figure 1B). Key serotypes (K1, K2, K5, K20, K54, K57) co-associate with virulence genes (*rmpA*, *rmpA2*, *iucA*, *iroB*, *peg-344*, and *wzy*(K1)). K1 and K2 are most studied, with *magA* specific to K1 and *rmpA*/*rmpA2* detected in nearly all K1 (99.4%) and K2 (98.6%) isolates, contributing to high lethality and serum resistance, respectively (Tian et al., 2025).

### Iron acquisition systems

Iron, though essential for bacterial metabolism, is sequestered by host proteins. *K. pneumoniae* produces siderophores to scavenge iron, crucial for infection survival (Schalk, 2025). All strains possess the chromosomal enterobactin (Ent) system with receptor *fepA* (Baghal et al., 2010). The chromosomal yersiniabactin (Ybt) system contributes to pathogenicity. Plasmid-borne aerobactin (*iucABCD-iutA*) and salmochelin (*iroBCDN*) systems strongly associate with hvKp (Holt et al., 2015). Amongst *iro* genes, *iroN* and *iroB* support efficient iron uptake (Müller et al., 2009), while *kfu* and *tonB* promote fitness and virulence (Hsieh et al., 2008) (Figure 1C). In hvKp, the siderophore biosynthesis gene *entC* is regulated by Ferric-uptake regulator (Fur) and RcsAB complex. Fur repression is relieved and RcsAB activates *entC* transcription under iron limitation, enhancing siderophore production and virulence (Yuan et al., 2020).

### Genotoxin

An important virulence factor in *K. pneumoniae* is colibactin, a genotoxin first identified in *Escherichia coli* (Choby et al., 2020). Colibactin induces DNA damage, causing cell cycle arrest, senescence, or apoptosis, impairing infection resolution (Faïs et al., 2018). It disrupts epithelial barriers, facilitates tissue translocation, and modulates immunity by reducing pro-inflammatory signals (Lu et al., 2017). Synthesised by nonribosomal peptide synthetases encoded in the *pks* locus, typically within a chromosomal ICE. ICE facilitates horizontal transfer of colibactin-associated virulence.

### Other virulence genes

Experimentally validated genes include *peg-344*, *mrk* fimbriae, *moaR* and *kva15* regulators, *kvgAS* signalling, and allantoin metabolism (Lai et al., 2003; Chou et al., 2004; Tu et al., 2009; Bulger et al., 2017). *ArcZ*, a small RNA regulator, represses virulence genes (Wu et al., 2024). CRISPRi screening identified cell envelope genes (*tolB, tolR, pal, lpp, ompA, waaL, nlpI*) contributing to virulence through maintaining membrane stability (Zhu et al., 2023).

## Plasmid-mediated dissemination of hypervirulence in *Klebsiella pneumoniae*

The clinical significance of virulence plasmids was first recognised when Nassif et al. identified a 180 kb plasmid encoding aerobactin and the mucoid phenotype, correlating with virulence phenotypes in K1 and K2 isolates (Nassif and Sansonetti, 1986). Subsequent epidemiological studies demonstrated global dissemination (Struve et al., 2015; Lei et al., 2024). Struve et al. showed that all 30 K1/K2 hvKp strains from patients with liver abscess or community-acquired pneumonia across seven countries (Africa, Asia, Europe, North America) during 1996–2012 harboured pLVPK-like plasmids, though some contained gene deletions (Struve et al., 2015). Additional virulence plasmids underscore their global spread and evolutionary significance. For example, pVir_030666 in *Klebsiella variicola* encodes multiple virulence determinants including mucoid phenotype regulators (*rmpA*, *rmpA2*), aerobactin (*iucABCD-iutA*), salmochelin (*iroBCDN*), and yersiniabactin (*irp1-2*, *ybtAEPQSTUX*), exhibiting enhanced virulence in larval infection models (Lu et al., 2018).

Most plasmid-mediated virulence in *K. pneumoniae* is largely driven by this conserved set of genes that enhance capsule formation, iron acquisition, and metabolic fitness. As discussed in the previous part, *rmpA* and *rmpA2* play central roles in increasing transcription of the *cps* locus, leading to the hypermucoviscous phenotype (Hu et al., 2023). This thick capsule protects the bacterium from complement-mediated killing and phagocytosis, enabling invasive disease even in healthy hosts.

In parallel, plasmid-encoded siderophore systems such as aerobactin (*iucABCD*-*iutA*) and salmochelin (*iroBCDN*) provide high-affinity mechanisms for iron uptake, allowing the pathogen to overcome host nutritional immunity and sustain rapid growth during infection (Hong et al., 2024).

Additional loci, including *peg-344* and plasmid-associated variants of the yersiniabactin cluster, further enhance fitness and facilitate tissue invasion. Together, these virulence determinants act synergistically to promote virulence plasmid dissemination, persistence, and severe clinical manifestations.

### Classical non-conjugative virulence plasmids

Classical virulence plasmids have been systematically classified by genetic architecture and virulence gene content. The two predominant types are KpVP-1, characterised by *iuc1*, *iro1*, *rmpA*, and *rmpA2*, and KpVP-2, carrying *iuc2*, *iro2*, and *rmpA* (Struve et al., 2015). pK2044 (224,152 bp), originally identified in a K1 hvKp strain, is the prototypical representative of the KpVP-1 lineage, whereas pLVPK (219,385 bp), first described in a K2 strain, is the representative plasmid of the KpVP-2 group. These large plasmids harbour key virulence determinants including aerobactin synthesis genes (*iuc*), metabolite transporter *peg-344*, and mucoid phenotype regulators *rmpA* and *rmpA2*. Loss of these plasmids significantly attenuates virulence in animal models (García-Cobos et al., 2025). Genomic surveillance revealed considerable diversity beyond classical archetypes. Novel variants include plasmids carrying *iuc3*, *iuc5* (with or without *iro5*), and novel *iuc*/*iro* allelic variants, demonstrating ongoing evolution and horizontal transfer across *K. pneumoniae* populations. Divergent virulence plasmids like pKP35_vir and pKP36_vir, sharing limited sequence homology (<40% coverage) with KpVP-1 and KpVP-2, have been designated KpVP-3, expanding recognised diversity (Struve et al., 2015).

### Conjugative virulence plasmids

**Figure 2:**
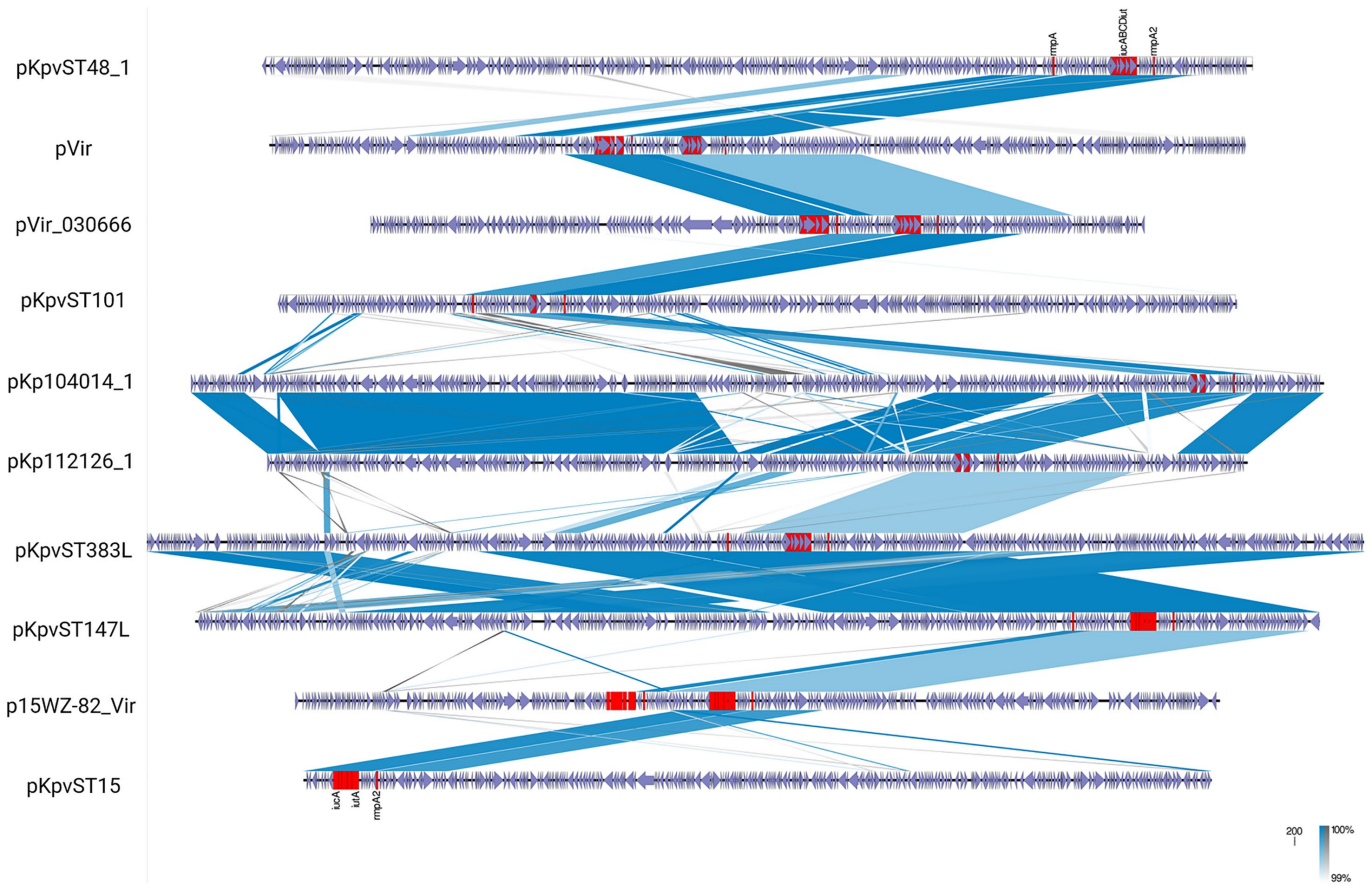
Comparative genomic alignment of representative conjugative virulence plasmids in *K. pneumoniae*. Linear comparison of virulence plasmids from *K. pneumoniae* reveals extensive sequence homology and structural rearrangements. Blue shaded areas represent shared regions with ≥99% nucleotide identity, with colour intensity reflecting sequence similarity. Arrows indicate predicted coding sequences, with virulence-associated genes (e.g., *iuc, iro, rmpA, rmpA2, peg-344*) highlighted in red. The figure is created by EasyFig (2.2.5).

A critical evolutionary development is the emergence of conjugative virulence plasmids, combining self-transmissibility with virulence gene cargo, enabling horizontal dissemination across bacterial populations. The first characterised conjugative virulence plasmid, p15WZ-82_Vir, was identified in *K. variicola* and formed through integration of a 100-kb virulence region into a conjugative IncFIB backbone. This chimeric plasmid retained key virulence loci including *rmpA*, truncated *rmpA2* (*rmpA2Δ*), aerobactin operon (*iucABCD-iutA*), and salmochelin cluster (*iroBCDN*). Experimental conjugation assays demonstrated successful transfer to multiple *Klebsiella* species, with transconjugants exhibiting significantly enhanced virulence, confirming both mobility and functional virulence contribution (Yang et al., 2019). Table 2 summarises ten individual conjugative virulence plasmids reported in previous studies. These plasmids were described as specific, well-characterised examples of plasmid-mediated hypervirulence (Table 2; Figure 2).

**Table 2:** Summary of conjugative virulence plasmid in *K. pneumoniae*.

Plasmid	Size	Siderophore genes	Capsule production gene	AMR Genes	Plasmid replicon type	Transferability	Accession number of plasmid	Bacteria host	ST of bacteria host	Serotype of bacteria host	Year	Country of isolation	Ref
p15WZ61-Vir	228,722	*iutA, iucABCD, iroCN*	*rmpA*	*bla*_KPC-2_, *bla*_CTX-M-65_, *foxA*, *bla*_TEM-1B_ and *catA2*	IncFIB	Not mentioned	ON777847.1	*K. variicola* 15WZ-61	ST11	K47	2024	China	https://doi.org/10.1016/j.micres.2024.127896
pKp104014_1	345,775	*iucABCDiutA*	*rmpA2*	*bla*_CTX-M-15_ *, bla*_TEM-1_ *, aac3’-IIa, dfrA1, sat2, bla*_SHV-5_ *, sul1, aadA1*	IncHI1B/IncFIB	IncFII *tra*	CP034046.1	*K. pneumoniae* KP_104014	15	K24	2014	Norway	https://doi.org/10.1093/jac/dkz028
p15WZ-82_Vir	292,280	*iroBCDNiucABCDiutA*	*rmpArmpA2*	-	IncHI1B/IncFIB	Not mentioned	NZ_CP032356.1	*K. variicola* 15WZ-82	595	KL16	2015	China	https://doi.org/10.1038/s41564-019-0566-7
pKp112126_1	299,188	*iucABCDiutA*	*rmpA2*	*bla*_TEM-1_ *, aac3’-IIa*	IncHI1B/IncFIB	IncFII *tra*	CP034054.1	*K. pneumoniae* KP_112126	15	K24	2015	Norway	https://doi.org/10.1093/jac/dkz028
pKpvST147L	343,282	*iucABCDiutA*	*rmpA, rmpA2*	*bla*_NDM-1_ *, armA, aph (3c)-VIb, sul1, sul2, mph(A), dfrA5*	IncHI1B/IncFIB	*tra*	NZ_CM007852.1	*K. pneumoniae* KpvST147L_NDM	147	KL35	2016	UK	https://doi.org/10.1099/jmm.0.000653
pKpvST15	277,162	*iucABCDiutA*	*rmpA, rmpA2*	*aac(6′)-Ib3, rmtC, bla*_CMY-6_ *, aac(6′)-Ib-cr, sul1*	IncHI1B/IncFIB	*tra*	NZ_CP040595.1	*K. pneumoniae* KpvST15_NDM	15	KL112	2016	UK	https://doi.org/10.3390/microorganisms7090326
pKpvST101	292,699	*iucABDiutA*	*rmpA, rmpA2*	*aph(6)-Id, aph(3′)-Ib, bla*_TEM-1B_ *, mph(A), sul1, sul2, dfrA5*	IncFII/IncFIB	*tra*	CP031369.2	*K. pneumoniae* KpvST101_OXA-48	101	KL62	2018	UK	https://doi.org/10.3390/microorganisms7090326
pKpvST48_1	302,220	*iroBCDNiucABCDiutA*	*rmpA, rmpA2*	*bla*_NDM-5_ *, bla_CTX-M-15_, bla*_OXA-9_ *, bla*_TEM-1B_ *, aph(3′)-Ia, aph(3′)-VIaac(6′)-Ib, aac(6′)-Ib-cr, aadA1, qnrS1, catA1*	IncHI1B/IncFIB	*tra*	CM016731.1	*K. pneumoniae* KpvST48_NDM	48	KL62	2018	UK	https://doi.org/10.3390/microorganisms7090326
pKpvST383L	372,826	*iroBCDNiucABCDiutA*	*rmpA, rmpA2*	*bla*_NDM-5_ *, bla*_CTXM-15_ *, bla*_OXA-9_ *, bla*_TEM-1B_ *, armA, dfrA5, aadA1, aph(3′)-1a, aph(3′)-VI, aac(6′)-lb, aac(6′)-lb-cr, catA1, sul1, sul2, mph(A), mph (E), msr(E), qnrS1*	IncHI1B/IncFIB	*tra*	CP034201.2	*K. pneumoniae* KpvST383_NDM_OXA-48	383	KL30	2018	UK	https://doi.org/10.3390/microorganisms7090326
pVir	297,984	*iroBCDN, iucABCDiutA*	*rmpA, rmpA2*	–	IncHI1B/IncFIB	*tra*	CP023723	*K. pneumoniae* TVGHCRE225	11	K47	2014	China	https://doi.org/10.1093/jac/dky164
pVir_030666	236,355	*iroBCDNiucABCDiutA*	*rmpA, rmpA2*	–	IncHI1B/IncFIB	*tra*	CP027063	*K. variicola* WCHKV030666	595	K16	2016	China	https://doi.org/10.1093/jac/dky301

### Emerging conjugative variants

Most virulence plasmids in *K. pneumoniae* are nonconjugative, lacking essential plasmid-transfer genes. However, compelling evidence exists for horizontal transfer between plasmids and chromosomes through various mechanisms (Nguyen et al., 2024). Structural complexity is exemplified by pLVPK, containing 13 ISs, likely representing sequential acquisition of horizontally transferred genes (Chen et al., 2004). Huang et al. described a unique virulence plasmid carrying *ybt4*, the only reported plasmid-encoded yersiniabactin locus (Huang et al., 2023). This 165-kb IncFIBκ/FIIκ plasmid contains a *tra-trb* conjugation region, and in pKP35_vir, the *ybt4* locus is flanked by mobile elements IS1 and Tn*2*, suggesting spread via genetic recombination (Huang et al., 2023). The molecular mechanisms governing virulence plasmid conjugation and stability remain incompletely characterised (Shon and Russo, 2012), necessitating comprehensive genomic characterisation of plasmid architecture, including core virulence gene cassettes and MGEs, to understand pathogenesis and develop surveillance frameworks (Ahmed et al., 2021).

## ICEs as drivers of *Klebsiella pneumoniae* virulence dissemination

ICEs are MGEs that integrate into bacterial chromosomes and transfer themselves through conjugation. ICEs consist of cargo modules and functional conjugation systems. Cargo genes, unrelated to their maintenance, provide selective advantages such as antimicrobial resistance genes (ARGs), heavy metal tolerance, or enhanced metabolic capabilities (Johnson and Grossman, 2015). Several ICEs carry virulence-associated cargo genes, contributing to pathogenicity. For instance, *P. aeruginosa* pathogenicity island 1 (PAPI-1) is a functional ICE that mediate horizontal transfer of virulence traits and may enhance ecological fitness, allowing colonisation and adaptation to specific hosts or environments (Carter et al., 2010).

The widespread distribution of ICEs across bacterial populations has been revealed through large-scale genomic analysis, which identified over 300 putative ICEs across more than 1,000 bacterial genomes. ICEs are found in both pathogenic and non-pathogenic bacteria, with a broader distribution than conjugative plasmids (Guglielmini et al., 2011). ICEs typically integrate at tRNA loci, with one study reporting 73% of strains harboured ICE insertions at one or more of four asparagine tRNA genes (Marcoleta et al., 2016).

They were first characterised in *Enterococcus faecalis* during the late 1980s, revealing tetracycline resistance transfer without plasmids (Franke and Clewell, 1981). The ICE in *K. pneumoniae* (ICEKp), extensively characterised by Lin et al., is defined by biosynthetic genes for siderophore yersiniabactin (Lin et al., 2008). ICEKp1 spans approximately 76 kilobases and was identified in hvKp strain NTUH-K2044. ICEKp1 demonstrated substantially higher prevalence in hvKp (38/42) compared to cKp strains (5/32) (Lin et al., 2008). ICEKp1 was also detected in in several members of the *K. pneumoniae* species complex (Breurec et al., 2016). Recent studies show ICEs are particularly prevalent in *K. pneumoniae* but occasionally acquired by other Enterobacteriaceae (Putze et al., 2009; Paauw et al., 2010). In a large-scale genomic survey of 2,498 *K. pneumoniae* isolates, approximately 40% of ICEKp carried *ybt*, and around 14% carried *clb* (Lin et al., 2008).

ICEKp elements are characterised by a conserved backbone including P4-like integrase (*int*), the 29-kb *ybt* locus, and a ~ 14-kb mobilisation module encoding *xis*, *virB*-type IV secretion system (T4SS), *oriT*, and *mobBC* (Lin et al., 2008; Marcoleta et al., 2016). Fourteen distinct structural variants, designated ICEKp1 through ICEKp14, have been identified, each associated with specific lineages of yersiniabactin and colibactin loci (Table 3) (Lam et al., 2018). Distinct cargo gene clusters at the right end allow classification into these structural variants.

**Table 3:** Comparative features of ICEKp variants in *K. pneumoniae.*

ICEKp variant	Key virulence genes	Cargo region features	Associated *ybt* lineages	Presence of Zn^2+^ Mn^2+^ metabolism module (KpZM)	Prevalent STs/lineages	Mobilisation genes
ICEKp1	*ybt, iro, rmpA*	18 kb insertion homologous to pLVPK (*iro, rmpA*)	*ybt1* (e.g., ST23)	No	ST23 (hvKp)	Present
ICEKp2-9	*ybt*	Lineage-specific accessory genes	Varies; each ICEKp mostly linked to one ybt	No	Diverse	Present
ICEKp10	*ybt, clb*	51 kb *clb* locus (colibactin); often with KpZM	*ybt1, ybt12, ybt17*	Yes (in most strains)	ST23, ST258, other CG258	Present
ICEKp11-14	*ybt*	Lineage-specific accessory genes	Varies	Sometimes	Diverse	Present
ICEKp (rhinoscleromatis)	*ybt* (with *irp2* nonsense mutation)	Lacks mobilisation module	*ybt11*	Not reported	ST67 (rhinoscleromatis)	Absent
ICEKp (NCTC 11697)	Highly divergent *ybt*	Lacks *virB*-T4SS and *xis*	Highly divergent (>2%) *ybt*	Not reported	Unknown	Absent

Most ICEKp variants are associated with a single *ybt* lineage, suggesting co-evolution, while ICEKp10, harbouring the *clb* (colibactin) locus, is linked to multiple *ybt* lineages, indicating repeated acquisition events. The presence of *clb* has been strongly associated with enhanced pathogenicity in both hvKp and cKp backgrounds, particularly in severe invasive infections. In contrast to ICEKp1, which primarily harbours *ybt*, *iro*, and *rmpA*, ICEKp10 lacks these additional virulence genes but contributes to hypervirulence through colibactin-mediated genotoxicity. Phylogenetic analysis of *clb* sequences revealed three distinct lineages (*clb1, clb2A, clb2B*), each associated with a specific *ybt* lineage, suggesting independent acquisitions into ICEKp. Certain ICEKp variants lacking typical mobilisation machinery indicate evolutionary divergence or specialisation within specific lineages (Table 3) (Lam et al., 2018).

ICEs spread virulence or resistance traits by excising from the chromosome, forming a circular intermediate, and transferring single-stranded DNA to recipient cells through a T4SS (Lee et al., 2010). Similar to other ICEs, ICEKp integrates into the chromosome at a conserved tRNA-associated attachment site (attB) through an ICE-encoded integrase, usually a tyrosine recombinase (Guérillot et al., 2014). After transfer, the ICE is re-integrated into the recipient chromosome, allowing stable maintenance of the virulence locus. The capability of ICEKp to mobilise virulence-associated loci, including *ybt* and *clb*, along with metabolic modules facilitating bacterial nutrient uptake, indicates its critical role in shaping pathogenic potential across various clonal backgrounds.

## How do tiny DNA segments control bacterial virulence?

ISs are the simplest form of mobile elements, typically consisting of a transposase gene flanked by short, inverted repeats. Though small, they have significant functional impacts by disrupting genes, modifying promoter activity, or facilitating genomic rearrangements (Wei et al., 2025). In *K. pneumoniae*, IS element involvement in virulence has been demonstrated in recent years, particularly through disruption of capsule biosynthesis gene *wcaJ* (Wang et al., 2022). Hypervirulent isolates of ST23-K1 strain harbour IS elements, such as IS*Kpn26* or IS*Kpn74*, which insert within *wcaJ* and induce frameshift mutations. This insertion significantly diminishes CPS production (Wang et al., 2022). Furthermore, IS*5*/IS*Kox3* elements have been observed associated with key capsule synthesis genes (*wcaJ, wza, wzc*) in hvKp. This association leads to non-mucoid phenotypes *in vitro*. Importantly, excision of these IS elements restores capsule production *in vivo*, thereby recovering hypervirulence capacity (Wei et al., 2025). The specific IS elements associated with *K. pneumoniae* virulence are detailed in Table 4.

**Table 4:** Summary of insertion sequence in *K. pneumoniae*.

IS	Size	Group	Location	Inserted gene	Origin	*K. pneumoniae* strain	Type (capsular & ST)	Country of isolation	Reference	Year of isolation	Accession number of strain
IS*Kpn26*	1,196 bp	IS*5* group, IS*5* family	Chromosome	*wcaJ*	*bla*KPC-2 plasmid	C1356, C400, C4599	K1; ST23	China	https://doi.org/10.1128/spectrum.02400-22	2015	SAMN30432938 SAMN24256184 SAMN24256185
IS*Kpn74*	1,056 bp	IS*903* group, IS*5* family	Chromosome	*wcaJ*	–	C2768	K1; ST23	China	https://doi.org/10.1128/spectrum.02400-22	2017	SAMN30432939
IS*Ec36*	∼12 kbps	IS*2* group, IS*3* family	pC6395_2	*rmpA2* and *iutA-iucABCD*	pK2044-like plasmid	C6395	K47; ST11	China	https://doi.org/10.1016/j.ijantimicag.2024.107245	2019	SAMN32217889
IS*Kpn28*	1,096 bp	IS*481* family	Chromosome	*rmpA2* and *iutA-iucABCD*	pNDM-Mar-like-pK2044-like fusion plasmids	C6395	K47; ST11	China	https://doi.org/10.1016/j.ijantimicag.2024.107245	2019	SAMN32217889
IS*26*	820 bp	IS*6* family	Chromosome	*rmpA2* and *iutA-iucABCD*	pNDM-Mar-like-pK2044-like fusion plasmids	C6395	K47; ST11	China	https://doi.org/10.1016/j.ijantimicag.2024.107245	2019	SAMN32217889
IS*3000*	3,235 bp	Tn*3* family	pNDM-Mar	*bla*_NDM-1_	pNDM-Mar	–	K47; ST11	Russia	https://doi.org/10.1016/j.ijantimicag.2024.107245	2019	–
IS*Kpn74*	1,056 bp	IS*903* group, IS*5* family	Chromosome	*iroBCDN, iucABCD/iutA, rmpA/A2 and peg*	–	PBIO2030	ST420	Germany	https://doi.org/10.3390/ijms22179196	2021	ERP130248
IS*903B*	1,057 bp	IS*903* group, IS*5* family	Chromosome	*wbaZ*	an 149214-bp plasmid	135,077	K64; ST11	China	https://doi.org/10.1128/msphere.00518-22	2006	CP073290; CP073296
IS*903D*	–	–	p11492-vir-CTXM	*bla*_CTX-M-24_	–	Strains 11,492	K1; ST23	China	https://doi.org/10.1128/AAC.02273-18	2019	CP026021; CP026022
IS*R1*	777 bp	–	Chromosome	*GmlB* glycosyltransferase gene	–	BIDMC 7B and ABC152	KL107-like; ST258, K64; ST147	Poland	https://doi.org/10.3390/ijms21186572	2013	JCNG00000000.1; JACENF000000000
IS*Ecp1*	1,656 bp	IS*1380* family	Chromosome	*rmpA2*	pKPTCM-1	KPTCM	K1; ST15	China	https://doi.org/10.3389/fcimb.2022.984479	2022	SAMN28422337
IS*5*	1,195 bp	IS*5* group, IS*5* family	Chromosome	*wcaJ, wza, and wzc*	*E. coli*	NK01067	KL1; ST23,	China	https://doi.org/10.1186/s13073-025-01474-0	2025	PRJNA1135710
IS*Kox3*	1,316 bp	IS*L3* family	Chromosome	*wcaJ, wza, and wzc*	*Klebsiella oxytoca*	NK01067	KL1; ST23	China	https://doi.org/10.1186/s13073-025-01474-0	2025	PRJNA1135710

IS-mediated inactivation of capsule biosynthesis genes conferred lower fitness cost and enhanced conjugation frequency of a *bla*_KPC-2_ resistance plasmid (Wang et al., 2022). These findings highlight a dynamic IS-mediated “capsule ON–OFF–ON” mechanism that not only alters virulence but also promotes horizontal transfer of multidrug resistance in hvKp (Wei et al., 2025). For instance, IS*Kpn74* has been identified as a significant factor altering virulence through two distinct yet contrasting mechanisms. Typically, plasmid-associated, IS*Kpn74* is integrated upstream of *rmpA* and *rmpA2*, enhancing expression of virulence traits such as hypermucoviscosity and increased siderophore production (Huang et al., 2024). Conversely, another study identified IS*Kpn74* within the chromosomal framework of an ST20 isolate, where its insertion occurs between K and O antigen loci, suggesting potential disruption of capsule biosynthesis pathways (Eger et al., 2021).

Beyond influencing strain virulence, ISs significantly contribute to structural evolution of virulence elements by facilitating transfer between plasmids and chromosomes. Research shows IS elements like IS*Kpn28* and IS*26* are instrumental in forming large fusion plasmids, arising from recombination events between classical virulence plasmids (such as pK2044-like) and resistance plasmids (like pNDM-Mar-like) (Wang S. et al., 2024). These hybrid plasmids contain key virulence genes, including *rmpA2* and the *iucABCD-iutA* operon. Furthermore, IS-mediated integration of these genetic fragments into the chromosome has been documented (Tian et al., 2022; Wang S. et al., 2024).

## Additional mobile elements: transposons and integrons

Transposons mobilise large genetic regions, including virulence cassettes, facilitating horizontal transfer across strains. The rapid evolution of CR-hvKp stems from transposon-mediated co-selection and co-transfer of virulence and resistance determinants (Gray et al., 2024). Tn*3* family members frequently mobilise virulence genes in *K. pneumoniae* (Tian et al., 2023). A conserved ~16.2 kb composite transposon on IncFIB/FII plasmids carries the *iuc3* aerobactin operon, enabling dissemination across animal and human reservoirs (Kaspersen et al., 2023). Tn*7074*-like transposons integrate complete virulence cassettes (*rmpA2, iucABCD-iutA, peg-344*) into conjugative plasmids, significantly enhancing recipient strain virulence (Li et al., 2025).

Integrons capture, assemble, and express gene cassettes encoding virulence factors (Chen et al., 2018). Integrons lack self-mobility and rely on ISs, transposons, or plasmids for dissemination (Farajzadeh Sheikh et al., 2024). Integrons, particularly class 1 integrons, are capable of shaping *K. pneumoniae* virulence potential (Chen et al., 2018). Studies found 74% of clinical isolates harboured class 1 integrons, with higher *wcaG* capsule gene prevalence (Derakhshan et al., 2016). Another study revealed 19.1% of integrons co-localised with virulence loci, including *iucABCD-iutA*, *rmpA2*, and *peg-344* (Li et al., 2025).

## MGEs-driven convergence of hypervirulence and resistance

CR-hvKP strains exhibit both hypervirulence and high-level AMR, spreading globally and presenting significant clinical threats (Karampatakis et al., 2023; Chen et al., 2020; Ahmed et al., 2021; Chen et al., 2021). An ST11 *K. pneumoniae* strain gained virulence after acquiring pLVPK-like plasmid pVir-CR-hvKP4, despite a 41,231-bp deletion including *rmpA* and *iro* loci (Gu et al., 2018). Salmochelin (*iro*) appears non-essential for systemic infection (Russo et al., 2015), and *rmpA*/*rmpA2* redundancy maintains pathogenicity (Cheng et al., 2010; Russo et al., 2015). Aerobactin production was predicted to be essential for hypervirulence (Russo et al., 2014).

There is evidence of harbouring hybrid plasmid pVir (297,984 bp), combining sequences from virulence plasmids pK2044/pLVPK and resistance plasmid pPMK-NDM in ST11 strain from Taiwan (Fang et al., 2025). Despite harbouring *iroBCDN*, *iucABCD-iutA*, *rmpA*, and *rmpA2*, it showed limited virulence in mouse models, suggesting unidentified virulence factors exist in the truncated regions from pK2044/pLVPK.

Tn*3*-family transposons mediate fusion between virulence and resistance plasmids, creating self-transmissible hybrids encoding both determinants (Ramirez et al., 2014; Nicolas et al., 2015; Tian et al., 2023). Integrons within multidrug-resistant IncFII plasmids, flanked by IS*26*, facilitate simultaneous horizontal transmission of resistance and virulence genes (Li et al., 2025).

## Concluding remarks and future directions

*K. pneumoniae* exemplifies a high-risk pathogen combining AMR and hypervirulence through MGEs acquisition, threatening both immunocompromised and healthy populations globally (García-Cobos et al., 2025). Despite extensive research on resistance mechanisms, critical knowledge gaps persist in the current understanding of this organism: studies remain confined to limited clonal groups and serotypes (Wyres et al., 2019); the hypervirulence-resistance relationship is underexplored (García-Cobos et al., 2025); virulence gene functions require deeper characterisation (Derakhshan et al., 2016); and MGEs-mediated mobility mechanisms remain incompletely understood (Haudiquet et al., 2021).

While over 200 published reviews have examined *K. pneumoniae* virulence since 1977, few address how virulence traits are acquired, mobilised, and maintained molecularly (Russo and Marr, 2019; Yang et al., 2021). Critically, plasmid-chromosome exchanges and smaller mobile elements (IS*Kpn74*, Tn*3* transposons) facilitating gene dissemination have received insufficient scientific attention (Haudiquet et al., 2021).

Understanding MGEs-mediated virulence dissemination is essential for advancing surveillance and therapeutics. Characterisation of MGE-mediated virulence gene mobility, including the roles of integrases, transposases, insertion sequences, and recombination hotspots, can enhance molecular diagnostic platforms by enabling the detection of both established virulence markers (such as *rmpA*, *iucABCD*, and *clb*) and their associated genetic mobility signatures (Farajzadeh Sheikh et al., 2024). Such integrated surveillance approaches would improve the identification of high-risk hypervirulent strains with enhanced horizontal transfer potential, thereby informing more effective infection control measures by distinguishing between clonal dissemination and independent MGE-mediated acquisition events across diverse lineages (Farajzadeh Sheikh et al., 2024). Furthermore, elucidating the molecular mechanisms governing ICE excision, plasmid conjugation, and IS-mediated genomic rearrangements may reveal novel therapeutic targets, including MGE-encoded integrases, relaxases, and T4SS components, whose inhibition could disrupt the horizontal spread of hypervirulence and AMR determinants (Wang Q. et al., 2024). Given the convergence of hypervirulence and carbapenem resistance traits mediated by hybrid plasmids and composite transposons, targeted disruption of MGE mobility machinery represents a promising strategy to limit the emergence and dissemination of CR-hvKp strains, which pose formidable challenges to global public health.

## Supplementary material

SUPPLEMENTARY FIGURE 1 Mechanism of ICE excision, transfer, and integration between bacteria. ICEs are found integrated in the donor chromosome at a specific attachment site (*attB*). Upon activation, ICEs excise via an integrase and recombination directionality factor (RDF/xis), forming a circular DNA intermediate. A relaxase introduces a nick at the origin of transfer (*oriT*) and facilitates the transfer of single-stranded DNA through a type IV secretion system (T4SS) into the recipient cell. Following transfer, the ICE is recircularised, undergoes second-strand synthesis, and is integrated into the recipient chromosome at an *attB* site by the integrase, completing the horizontal transfer process. The figure is created by BioRender.
