# Supplementary figures and images for: Mobile genetic elements in shaping Klebsiella pneumoniae pathogenicity
Source: Front Microbiol. 2026 Jan 6;16:1730961. doi: 10.3389/fmicb.2025.1730961 (PMC12816380; doi:10.3389/fmicb.2025.1730961)

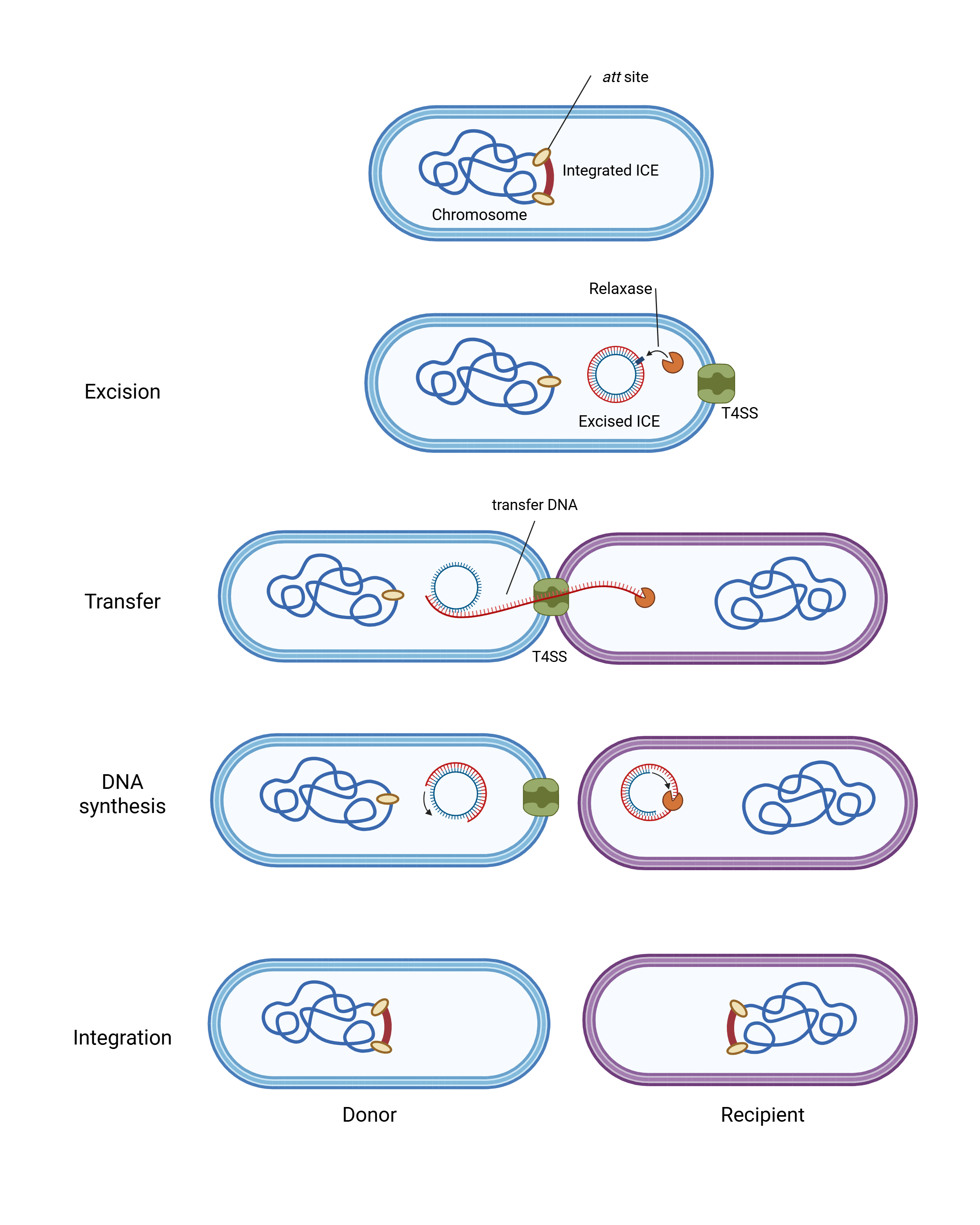

Supplement: Supplementary file 1 [file Image_1.tif]
